# Radiation Risks and Interventional Cardiology: The Value of Radiation Reduction Exposure

**DOI:** 10.3390/jcdd10030121

**Published:** 2023-03-14

**Authors:** Maria Grazia Andreassi

**Affiliations:** CNR Institute of Clinical Physiology, 56124 Pisa, Italy; mariagrazia.andreassi@cnr.it; Tel.: +39-0503152628

Fluoroscopically guided cardiac procedures are an essential component of care in the practice of cardiology, and are, in most cases, lifesaving.

However, exposure to ionizing radiation during cardiac fluoroscopic interventions has become a topic of growing concern due to increasing volumes, the complexity of certain procedures, and the cumulative exposure from multiple tests [1]. 

Indeed, it is not infrequent for patients to undergo recurrent diagnostic and therapeutic ionizing fluoroscopic procedures, exceeding the dose equivalent of 300–1000 chest X-rays in each exam, increasing the cumulative dose and cumulative risk of adverse effects over a lifetime. 

Similarly, the operator dose per procedure correlates somewhat with the patient dose due to their working position being close to the X-ray beam and the patient (the source of scatter radiation).

Given their chronic radiation exposure in cardiac catheterization laboratories, interventional cardiologists are among the most highly exposed physicians [2,3]. 

Ionizing radiation exposure may adversely impact both patients and medical personnel involved in fluoroscopic procedures. It is known that the deleterious effects of ionizing radiation include deterministic and stochastic effects. Deterministic effects (e.g., erythema or skin burns) are dose-dependent and occur after a specific threshold of radiation exposure, below which the outcome is not observed. Some interventional procedures with a long duration and multiple image acquisition (e.g., percutaneous coronary intervention, radio-frequency ablation) may induce deterministic effects in both staff and patients [1,3,4].

The stochastic effects are dose-independent and have a longer latency period than the deterministic effect. The main stochastic risks of radiation exposure are cancer and genetic effects. 

Radiation exposure is particularly important for pediatric patients because they are more vulnerable to the oncogenic effects of radiation and the lifetime cancer risk might be 2–3 times higher than the estimates for older populations [5].

In recent years, clinical and epidemiological studies in the cardiology population have supported the possibility of increased cancer risk derived from cardiac fluoroscopic catheterization [6], especially in patients receiving cumulative radiation doses in their early lives [7].

Furthermore, there is evidence of a potential increased risk of cancer in interventional cardiologists in highly exposed organs, such as the brain and skin [3,8].

More recently, there has been also considerable interest in the potential non-cancer diseases at low and moderate doses in the scientific community, in particular regarding the risk of cardiovascular disease and cognitive and neurodevelopmental effects [3,9,10]. 

However, the low dose radiation-related risks of cancer and non-cancer are still uncertain and, therefore, more research is essential for providing relevant information for radiation protection and for understanding the biological mechanisms [2,7]. 

In any case, every effort should be made to minimize the radiation dose to the patient and decrease the scatter dose to the operator by using techniques that reduce the dose and maintain image quality. 

In a recent study, Wang et al. demonstrated the importance of radiation-sparing practices, which are able to substantially decrease radiation exposure in patients [11].

The authors showed that reducing the frame rate are able to directly decrease the radiation dose received by patients without affecting the success rate [11].

Previous studies showed that a frame rate-lowering strategy offers a simple and very effective measure to reduce patient radiation exposure without compromising the quality of imaging and patient outcomes [11,12].

Additionally, Wang et al. showed that wearing protective equipment significantly reduced radiation exposure in radiosensitive organs (eye, thyroid, and reproductive organs) without interfering with procedure performance. This study’s findings demonstrate that the use of proper radiation protection equipment is a promising method for decreasing harm caused by the main radiation beam located under the table and the scattered radiation from all directions.

Additionally, the percutaneous transcatheter radiofrequency ablation of atrial fibrillation with remote-controlled magnetic navigation has recently been shown to provide comparable success rates with reduced fluoroscopy time and radiation exposure for both patients and physicians as compared to conventional manual ablation techniques [13,14].

These data represent a convincing argument for the importance and value of education and awareness for the operator to optimize procedures and enhance the radiation safety of cardiac fluoroscopic interventions and imaging [15]. There is no doubt that radiological and nuclear medicine examinations are the cornerstone in cardiology. However, the radiation risks are not negligible and multiple examinations lead to a substantial cumulative radiation dose in the individual patient [16].

The education and promotion of radiation safety among cardiologists is critical for ensuring adherence to new protocols, which significantly reduces radiation exposure to the patient, the interventional cardiologist, and the catheterization laboratory staff [1,15].

Moreover, education is essential to limit unnecessary testing and encourage the use of an alternate imaging modality, when the clinical information can be obtained with a non-ionizing test with comparable accuracy [15].

Finally, the radiation issue in cardiology warrants further research to understand the long-term impact of risks, such as cancer and non-malignant diseases.
